# Electrical Treatment in Salpingitis

**Published:** 1888-04

**Authors:** 


### ELECTRICAL TREATMENT OF SALPINGITIS.

At the meeting of the Société de Médecine, of Paris, on February 11, M. APOSTOLI read a paper on this subject, in which he drew the following conclusions :

1. The fever and inflammatory state are not an absolute indication in gynæcology to the methodical and appropriate application of the electric current.

2. Non-inflammatory suppurations of the uterus may be advantageously treated by the constant current, which, if it is favorable in congestive periods and inflammations of the first degree, appears to me, nevertheless, to be contra-indicated in cases of confirmed suppuration. I make an exception of the cases in which electric cauterization, in the tubular form, serves to create with the pus, near the vaginal wall, a more favorable and surer tissue.

3. The penetrating galvano-caustic, in the form of galvano-puncture, is a precise method that may serve a double purpose : (*a*) To absorb a phlegmasia at the beginning, and to arrest an inflammatory process in its evolution. (*b*) To permit easy evacuation of a fluid collection through the channel made by the puncture, provided this collection is in the vaginal cul-de-sac.

4. Every inflammatory exudation occurring in the vaginal cul-de-sac may be justifiably treated by the penetrating galvano-caustic (under restrictions to be mentioned).

5. This method may be applied with success to certain cases of salpingitis and hydro-salpingitis, and with so much the more ease and harmlessness as the tumor is nearer the vaginal wall.

6. In every galvano-puncture the operator should rigidly observe the rules already laid down concerning the site of puncture, its depth, the size of the trocar, antiseptic precautions, the rest of the patient, etc.

7. Only two negative vaginal galvano-punctures, in a case of acute hydro-salpingitis, are sufficient to cause very rapidly a considerable anatomical regression and an absolutely symptomatic cure.—*Gazette de Gynécologie,* March 1, 1888.

